# The impact of Mild Behavioral Impairment on the individual’s level of psychological, social, and occupational functioning

**DOI:** 10.1192/j.eurpsy.2022.457

**Published:** 2022-09-01

**Authors:** C. Elefante, G.E. Brancati, T. Gemmellaro, S. Ricciardulli, F. Romeo, S. Torrigiani, G. Pistolesi, S. Amadori, L. Lattanzi, G. Perugi

**Affiliations:** University of Pisa, Department Of Clinical And Experimental Medicine, Pisa, Italy

**Keywords:** Mild Behavioral Impairment (MBI), Neuropsychiatric symptoms, Preclinical dementia, global functioning

## Abstract

**Introduction:**

Mild behavioral impairment (MBI) is a neurobehavioral syndrome characterized by later-life emergent neuropsychiatric symptoms, which represent an at-risk state for incident cognitive decline and dementia.

**Objectives:**

Our obective was to prospectively evaluate the impact of MBI on global functioning in patients ≥ 50 years with a major depressive episode (MDE) at baseline.

**Methods:**

We recruited 51 patients ≥ 50 years presenting with a MDE at the outpatient clinic of the 2^nd^ Psychiatric Unit of the University of Pisa. Then we selected those patients who had a follow-up of at least two months and excluded subjects with a neurodegenerative disease. The included patients (N = 25) were subdivided in a subgroup with MBI and a subgroup without MBI. The subgroups have been compared for the difference between baseline and follow-up score in global functioning according Global Assessment of Functioning (GAF) scale. Comparative analyses were conducted by means of mixed anova.

**Results:**

There was a significant interaction effect between time and the MBI condition (F[1, 23] = 4.12, p = 0.05 η p 2 = 0.15). Descriptive statistics showed that while patients without MBI showed higher GAF score at follow-up (mean = 65.12) compared to GAF score at baseline (mean = 54.37), patients with MBI showed, on average, the same GAF score at follow-up (mean = 54.44) and at baseline (mean = 54.44).

**Conclusions:**

In patients with MDE, the presence of MBI is related to a lack of improvement in psychological, social, and occupational functioning in the short-term

**Disclosure:**

No significant relationships.

